# HIV viral transcription and immune perturbations in the CNS of people with HIV despite ART

**DOI:** 10.1172/jci.insight.160267

**Published:** 2022-07-08

**Authors:** Shelli F. Farhadian, Ofir Lindenbaum, Jun Zhao, Michael J. Corley, Yunju Im, Hannah Walsh, Alyssa Vecchio, Rolando Garcia-Milian, Jennifer Chiarella, Michelle Chintanaphol, Rachela Calvi, Guilin Wang, Lishomwa C. Ndhlovu, Jennifer Yoon, Diane Trotta, Shuangge Ma, Yuval Kluger, Serena Spudich

**Affiliations:** 1 Department of Medicine, Section of Infectious Diseases, and; 2 Department of Neurology, Yale School of Medicine, New Haven, Connecticut, USA.; 3 Program in Applied Mathematics, and; 4 Interdepartmental Program in Computational Biology and Bioinformatics, Yale University, New Haven, Connecticut, USA.; 5 Department of Immunobiology, Yale School of Medicine, New Haven, Connecticut, USA.; 6 Department of Medicine, Division of Infectious Diseases, and; 7 Feil Family Brain & Mind Institute, Weill Cornell Medicine, New York, New York, USA.; 8 Department of Biostatistics, Yale School of Public Health, New Haven, Connecticut, USA.; 9 University of North Carolina-Chapel Hill, Chapel Hill, North Carolina, USA.; 10 Bioinformatics Support Program, Cushing/Whitney Medical Library, Yale School of Medicine, New Haven, Connecticut, USA.; 11 Yale Center for Genome Analysis, Yale University, New Haven, Connecticut, USA.; 12 Department of Pathology, Yale School of Medicine, New Haven, Connecticut, USA.

**Keywords:** AIDS/HIV, Neuroscience, Neurological disorders

## Abstract

People with HIV (PWH) on antiretroviral therapy (ART) experience elevated rates of neurological impairment, despite controlling for demographic factors and comorbidities, suggesting viral or neuroimmune etiologies for these deficits. Here, we apply multimodal and cross-compartmental single-cell analyses of paired cerebrospinal fluid (CSF) and peripheral blood in PWH and uninfected controls. We demonstrate that a subset of central memory CD4^+^ T cells in the CSF produced HIV-1 RNA, despite apparent systemic viral suppression, and that HIV-1–infected cells were more frequently found in the CSF than in the blood. Using cellular indexing of transcriptomes and epitopes by sequencing (CITE-seq), we show that the cell surface marker CD204 is a reliable marker for rare microglia-like cells in the CSF, which have been implicated in HIV neuropathogenesis, but which we did not find to contain HIV transcripts. Through a feature selection method for supervised deep learning of single-cell transcriptomes, we find that abnormal CD8^+^ T cell activation, rather than CD4^+^ T cell abnormalities, predominated in the CSF of PWH compared with controls. Overall, these findings suggest ongoing CNS viral persistence and compartmentalized CNS neuroimmune effects of HIV infection during ART and demonstrate the power of single-cell studies of CSF to better understand the CNS reservoir during HIV infection.

## Introduction

Over the last 30 years, breakthroughs in the successful treatment of HIV infection have radically improved the lives of millions of people living with HIV (PWH). PWH with access to combination antiretroviral therapy (ART) are now predicted to enjoy a life expectancy approaching that of the non–HIV-infected population, largely free of the devastating opportunistic infections that characterized life for PWH during the pre-ART era (1). However, PWH on suppressive ART remain at higher risk for numerous non-AIDS conditions when compared with the general population and display elevated systemic markers of immune-mediated inflammation (2–7). Viral eradication through HIV cure is therefore the ultimate treatment goal for PWH. However, an incomplete understanding of tissue viral reservoirs, including in the brain, remains a major impediment to HIV cure efforts.

Neurological abnormalities in PWH correlate both with measures of CNS viral persistence and with abnormal CNS immune cell activation, with some of these abnormalities enduring even in patients on suppressive ART (8–10). In the brain, persistent immune activation during virologically suppressed HIV infection is evident via neuroimaging and brain autopsy studies demonstrating neuroinflammation in individuals who die with HIV infection (11, 12). However, the specific CNS immune cell pathways that stay deranged in PWH remain unknown, and the identity of cells in the CNS compartment in which HIV may persist during ART remains elusive.

Cerebrospinal fluid (CSF) is the only CNS tissue routinely and safely accessible in living humans. As CSF bathes the brain and CNS, cells sampled through lumbar puncture provide a window into immune cells infiltrating the brain parenchyma, and transcriptomic studies of CSF cells have been used to assess the CNS immune environment during other neuroinflammatory conditions, including multiple sclerosis, Alzheimer’s disease, and COVID-19 (13–19). In HIV, prior studies of the CSF have primarily focused on soluble immune markers and demonstrate elevations in markers of lymphocyte and myeloid lineage activation in the CSF of PWH on ART compared with uninfected controls (9, 10, 20–22).

To better understand neuropathogenesis during long-standing ART-treated HIV infection, here we perform a high-resolution characterization of viral persistence and immune perturbations in the CNS using paired CSF and peripheral blood samples in PWH and matched uninfected controls. By assessing single transcriptomes for HIV-1 viral transcripts, we demonstrate HIV-1 viral persistence in single cells in the CSF at higher levels than found in the peripheral blood.

Our studies reveal that transcription of HIV-1 in central memory CD4^+^ T cells persists in the CNS of PWH despite ART and that Th1-mediated CD8^+^ T cell activation is a hallmark of the CNS immune response during chronic HIV infection. Taken together these findings demonstrate the utility of multimodal single-cell analyses to probe the CNS during chronic HIV infection.

## Results

### Characteristics of study participants.

Research participants consented to the Yale HIV Associated Reservoirs and Comorbidities (HARC) cohort study from January 2018 to March 2020. Community-dwelling PWH on suppressive ART were enrolled (*n* = 44), as well as HIV-uninfected control volunteers (*n* = 22). All enrolled participants consented to lumbar puncture and blood draw for research studies. Demographic and clinical characteristics of all participants are shown in Table 1. In the PWH, the median duration of viral suppression was 19 years, most participants (24/44) were on an integrase inhibitor–based regimen at the time of lumbar puncture, and all had suppressed viral loads in plasma (<20 copies/mL). For single-cell RNA sequencing, which requires freshly drawn CSF, a subcohort of 6 PWH and 4 matched uninfected controls were enrolled (Supplemental Table 1; supplemental material available online with this article; https://doi.org/10.1172/jci.insight.160267DS1). These participants underwent detailed neuropsychological testing, which did not reveal differences between the 2 groups (Supplemental Table 1).

### Distribution of immune cell populations in the CSF is markedly different from blood.

To differentiate immune cell changes occurring specifically in the CNS compared with the peripheral blood of PWH, we analyzed paired blood and CSF samples, collected simultaneously, and analyzed single-cell transcriptomes from PBMCs and CSF cells from PWH and uninfected controls.

**Figure 1:**
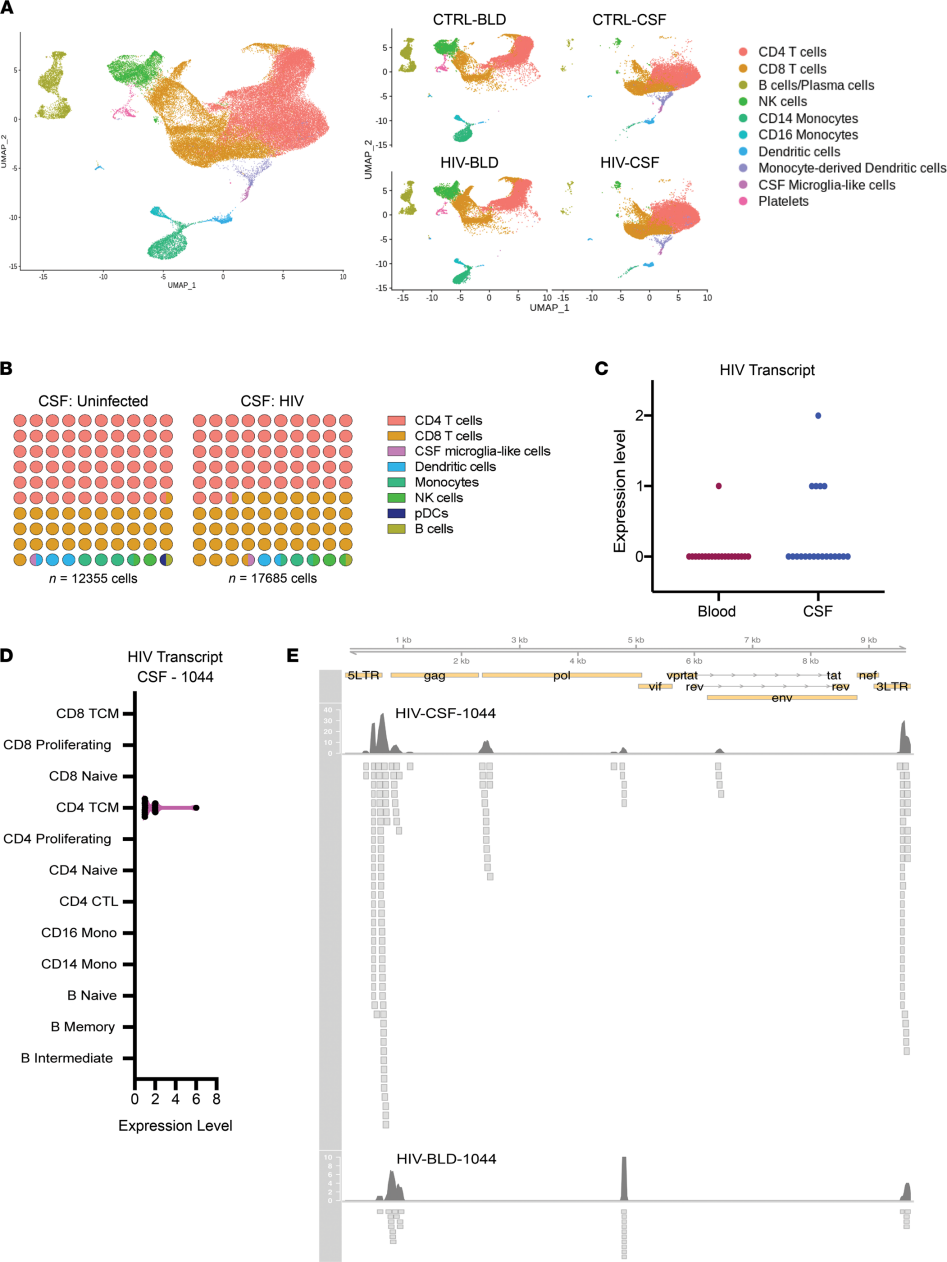
Single-cell RNA sequencing of CSF and blood from PWH and uninfected controls. (**A**) Combined (left) and split (right) uniform manifold approximation and projection (UMAP) of CSF cells and PBMCs (blood, BLD) from PWH and uninfected controls (CTRL). *n* = 75,734 cells (30,040 CSF cells and 35,694 PBMCs). (**B**) Frequency of immune cell types in the CSF in PWH compared with uninfected controls demonstrating significantly (*P* < 0.05, χ^2^ test) more CD8^+^ and fewer CD4^+^ T cells in the CSF of PWH compared with uninfected controls (*n* = 17,685 cells from 5 PWH and *n* = 12,355 cells from 4 uninfected controls). (**C**) HIV transcript expression levels in blood (PBMCs) and CSF single cells mapped to consensus HXB2 HIV reference sequence. (**D**) HIV transcript expression within annotated immune cell subsets using reference-based mapping. Shown in pink is violin plot with black dots representing individual cells in which HIV transcripts were detected. For **C** and **D**, data are combined from 4 PWH (*n* = 16,147 CSF cells, and *n* = 17,061 PBMCs). (**E**) Alignment track of HIV transcripts in all single cells from CSF and blood of 1 participant (HIV-1044) across the annotated HXB2 consensus sequence. Top panel with read coverage displayed as histogram and below is pile-up view of the individual reads. pDC, plasmacytoid DC; TCM, T central memory; CTL, cytoxic T lymphocyte.

CSF and PBMC single-cell transcriptomes were first combined, resulting in 75,734 cells (*n* = 30,040 CSF cells and *n* = 35,694 PBMCs). A median of 4964 cells and 4264 cells per sample were included from PBMCs and CSF, respectively. Upon principal component and unsupervised cluster analysis (23), these cells formed 10 distinct clusters (Figure 1A). We annotated these clusters using canonical immune marker genes (Supplemental Figure 1) and found that both CSF and blood contained major immune cell types, as expected, but that the distribution of immune cell populations in the CSF was markedly different from peripheral blood. When compared to the PBMCs, CSF showed an increased frequency of microglia-like cells (0.70% versus 0.0095%; *P* < 0.001), consistent with prior reports (13, 18, 24). This was true for CSF from PWH and from controls. CSF also contained increased frequency of dendritic cells (2.0% versus 0.2%; *P* < 0.001) and decreased frequency of B cells (0.5% versus 10.2%; *P* < 0.001) and NK cells (1.1% versus 11.7% *P* < 0.001) when compared with blood.

We next determined whether there were differences in the frequency of immune cell subsets in the CSF in PWH when compared with HIV-negative controls. We performed unsupervised cluster analysis of all CSF cells, revealing 8 clusters corresponding to known immune cell types (Figure 1B). CSF from PWH demonstrated an increased frequency of CD8^+^ T cells (42% versus 32%; *P* < 0.01) and a decreased frequency of CD4^+^ T cells (51% versus 59%; *P* < 0.01) compared with CSF from controls. There were no other significant differences in the frequencies of other major immune cell types in CSF when comparing PWH to controls using this cluster-based approach.

### HIV-1 polyA transcripts are detected in CSF cells more frequently than in the blood.

Our single-cell RNA-sequencing (scRNA-Seq) pipeline utilizes the 10x Genomics 3*′* Gene Expression assay, which captures host cellular and viral transcripts by reverse-transcribing polyadenylated mRNAs. Since HIV-infected cells contain polyadenylated HIV mRNAs, we probed the single-cell transcriptomes from CSF and blood to identify single HIV-infected cells by aligning the cellular sequencing reads to a custom reference with viral sequence HIV-1 HXB2 reference genome (K03455.1). We found rare cells producing HIV transcripts in the CSF of 4/4 and the blood of 1/4 PWH who underwent paired scRNA-Seq of blood and CSF (*P* < 0.03, χ^2^ test). (Figure 1C). We did not detect HIV mRNA transcripts in any samples from uninfected controls. To better characterize the cell types harboring HIV RNA transcripts, we next analyzed 6,066 PBMCs and 10,076 CSF cells derived from 1 PWH participant (HIV-1044) who was previously found to have low-level HIV RNA in CSF (23 copies/mL) despite ART. In this participant, 1 peripheral blood T cell and 15 CSF T cells were found to harbor HIV transcripts (*P* < 0.03, χ^2^ test), with all transcripts localized to central memory CD4^+^ T cells (Figure 1D). As expected from 10x Genomics single-cell reads and mapping to a consensus reference sequence, alignment of HIV transcripts in CSF and blood revealed biases toward the 3*′* and 5*′* long terminal repeat regions (Figure 1E).

### CD204 is a reliable marker of microglia-like cells from the CSF.

Microglia and CNS monocytes have been suggested to play a prominent role in HIV neuropathogenesis (11, 25–27). However, myeloid cells in the CSF are challenging to analyze since their scarcity makes it difficult to characterize cell subsets through traditional flow cytometry–based methods. We used cellular indexing of transcriptomes and epitopes by sequencing (CITE-seq) (28), a single-cell phenotyping method that uses DNA-barcoded antibodies to tag individual cells that are then processed for scRNA-Seq, and which can characterize even rare cell types according to cell surface proteins. We previously reported the presence of a rare (<5%) population of cells in the CSF that demonstrate a gene expression profile similar to brain microglia, are found almost exclusively in CSF compared with blood, and are more abundant in the CSF of PWH compared with the CSF of uninfected controls (24). We used custom-made CITE-seq antibody-oligonucleotide conjugates to determine the surface cell marker expression in these CSF microglia-like cells. Our panel included traditional markers of monocyte subsets (CD163, CD14, CD16, CD1C, CD11b), and CD204, since our previous transcriptome analysis of the cluster of microglia-like cells in the CSF suggested that the *MSR1* gene, which encodes the cell surface protein CD204, was enriched in this group of cells (24). Prior neuropathology studies have demonstrated CD204 protein expression in activated microglia and CNS perivascular macrophages, but it was unknown whether this protein is expressed on CSF microglia-like cells (29, 30).

**Figure 2:**
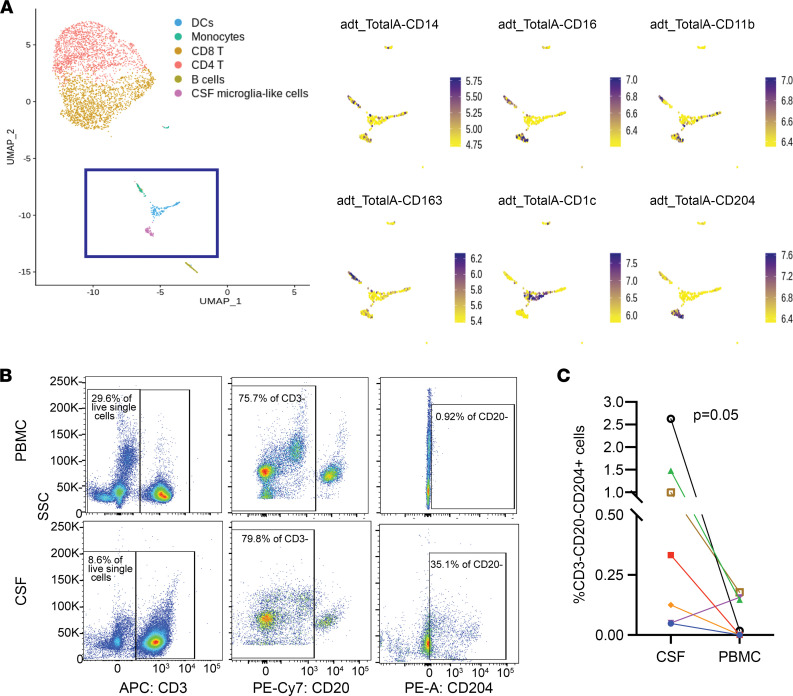
CD204 is a protein marker for CSF microglia-like cells. (**A**) Combined protein and transcriptome analysis of CSF cells in a person with HIV. Left panel shows clustering of single cells based on transcriptome analysis. Myeloid cells are contained within the purple box. Right panel shows CITE-seq analysis of 6 protein markers of CSF myeloid cells (UMAP), demonstrating that CD204 is a protein marker for CSF microglia-like cells. (**B**) Representative flow cytometry plot and (**C**) frequency of CD3^–^CD20^–^CD204^+^ cells in the CSF and the PBMCs of PWH detected by flow cytometry. Each color represents an individual participant. *n* = 7. Two-sided *P* = 0.05, Wilcoxon matched pairs signed rank test.

CITE-seq analysis confirmed that CSF microglia-like cells, but not other CSF cell populations, expressed high levels of CD204 (Figure 2A and Supplemental Figure 2). We found variable protein expression of CD14 and CD16 in this cluster. We therefore used CD204 to phenotype CSF cells and PBMCs from PWH through traditional flow cytometry (Figure 2B). We found CD3^–^CD20^–^CD204^+^ cells in the CSF of 7 out of 7 PWH, and they comprised a mean 0.80% of all CSF cells (Figure 2C). In contrast, these cells were detected in 3 out of 7 PBMC samples and at much lower frequency (mean 0.07% of all PBMCs; *P* < 0.05). This suggests that CD204 is a reliable marker that may be used to isolate microglia-like cells from the CSF. Using scRNA-Seq analyses, we did not detect any HIV-1 RNA in any microglia-like cells in any of the CSF samples we tested (Figure 1D).

### CSF T cells in PWH contain differentially abundant T cell subsets compared with uninfected controls.

Since the majority (>90%) of cells in the CSF are T cells, we next assessed for differences in T cell subsets between PWH and uninfected controls to identify immune perturbations associated with HIV. We used 2 computational approaches to identify CNS-specific T cell immune states: differential abundance analysis and feature selection using Stochastic Gates (STG), a potentially novel embedded feature selection method for supervised deep learning models designed to select gene subsets that lead to accurate (disease state) predictions.

While traditional unsupervised cluster analysis is useful to identify major cell subsets in scRNA-Seq data, cluster-based methodologies may be inadequate to detect subpopulations that are distinct between 2 groups if the subpopulations do not fall into well-defined clusters or are contained within part of a cluster. We therefore applied DA-seq, a multi-scale method to detect differentially abundant (DA) subpopulations that are distinct between 2 scRNA-Seq data sets (31). We used DA-seq to identify subpopulations of T cells that were more or less abundant in the CSF of PWH compared with controls.

**Figure 3:**
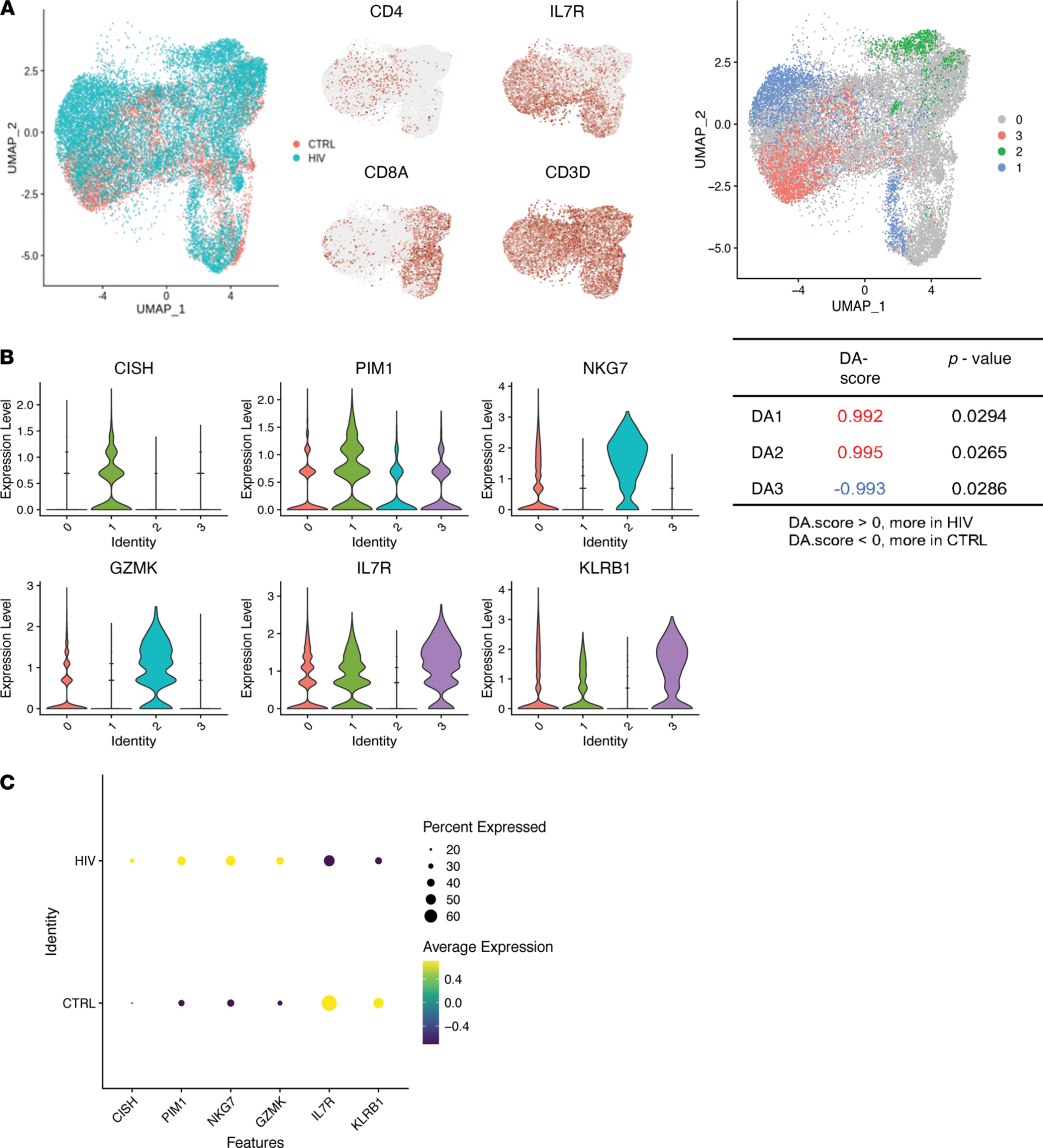
Differential abundance analysis of CSF T cells. (**A**) Left, UMAP projection of CSF T cells from PWH (blue) and uninfected controls (CTRL; red). Right, cells are colored if they belong to a region that is DA in PWH compared with CTRL. Identities 1 and 2 are DA regions that were found more frequently in PWH, and identity 3 is the DA region that was found less frequently in PWH compared with uninfected controls. Cells colored gray are not in DA regions. (**B**) Violin plot showing expression of genes that distinguish the DA regions from all other T cells. (**C**) Dot plot showing average expression of DA T cell marker genes in the CSF T cells of PWH compared with uninfected controls. The size of the dot corresponds to the percentage of cells expressing the gene. The color represents the average expression level. *n* = 5 PWH and *n* = 4 uninfected controls.

DA-seq applied to CSF T cells revealed 3 groups of cells that were found more (group 1 and 2) or less (group 3) frequently in PWH compared with uninfected controls (*P* < 0.05) (Figure 3A). Importantly, these subpopulations of T cells were not identified using traditional cluster-based approaches. DA group 1 consisted of a subpopulation of CD4^+^ T cells with high expression of *CISH* and *PIM1*, the latter of which has been shown to regulate human Th1 cell differentiation and play a role in immune cell activation and proliferation (32) (Figure 3, B and C). DA group 2, which consists of a small subpopulation of CD8^+^ T cells found almost exclusively in the CSF of PWH but not controls, showed high expression of cytotoxic genes NKG7 and GZMK (Figure 3, B and C). DA group 3 consisted of a group of CD4^+^ T cells found in uninfected controls but not in PWH, with high expression of IL7R (CD127) and KLRB1 (CD161), markers of IL-17–producing Th17 cells (33) (Figure 3, B and C).

### Machine learning classifies CSF and blood T cells from PWH and uninfected controls.

The results of differential abundance analysis suggested that standard clustering approaches are insufficient to detect gene expression changes in CSF T cells in PWH compared with controls. We therefore used feature selection using STG, a machine learning approach that identifies relevant combinations of features (in this case, combinations of genes) within a data set that, together, can classify high-dimensional data. We applied feature selection by STG to identify combinations of genes that, when evaluated together, could predict with high accuracy whether a T cell originated from a person with HIV versus from an uninfected control, thereby revealing combinations of genes that “mark” a cell as related to HIV.

**Figure 4:**
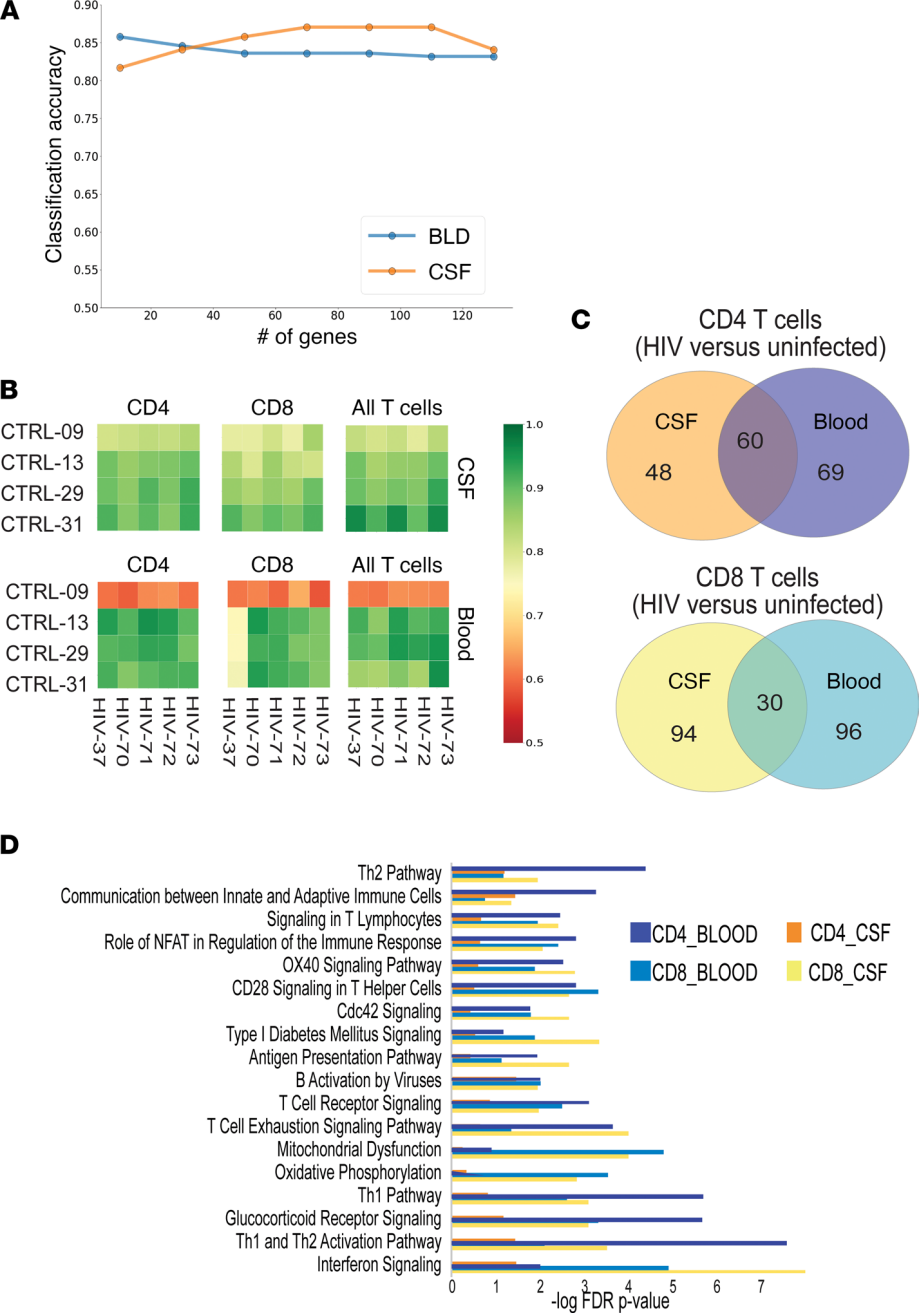
Feature selection by STG identifies genes that differentiate compartment (CSF or blood) and disease state (HIV or uninfected) with high accuracy. (**A**) Accuracy plots for predicting HIV versus uninfected donor status of CSF or blood cells using the number of features shown on *x* axis. (**B**) Accuracy heatmaps for individual test pairs using a leave-two-out cross-validation. Green indicates that the disease state (HIV or uninfected) of the sample was ascertained with high accuracy using genes derived from the STG model. (**C**) Venn diagrams for genes that predict disease state in each compartment and cell type. (**D**) Ingenuity pathway analysis (IPA) of genes from **C**. *n* = 5 PWH and *n* = 4 uninfected controls.

First, using STG, we found that the expression of 60 genes taken together could be used to predict the disease state (HIV or uninfected) of a peripheral blood sample with more than 81% average accuracy (Figure 4A, blue line) and that the expression of 120 genes taken together could be used to predict disease state (HIV or uninfected) of a CSF sample with more than 84% average accuracy (Figure 4A, orange line). We then validated these gene sets using a leave-two-out cross-validation procedure and found that the gene set STG identified was able to identify the donor’s disease status (HIV or uninfected) for CSF T cells at greater than 90% accuracy for all combinations of PWH and uninfected controls in our sample set (Figure 4B). In blood, we found that when 1 particular healthy control (participant 3009) was used in the training model, the accuracy of prediction fell to 60% and was otherwise greater than 80% for predicting disease status of a T cell donor.

We found that both shared and unique genes predicted disease status in CSF versus blood, for CD4^+^ and CD8^+^ T cells (Figure 4C). Ingenuity pathway analysis of these genes revealed that CD4^+^ T cells in the peripheral blood from PWH differed from CD4^+^ T cells in the peripheral blood from uninfected controls through the expression of genes related to T cell activation and exhaustion pathways, whereas gene expression in CD4^+^ T cells in the CSF of PWH was largely similar to gene expression in CD4^+^ T cells in the CSF of uninfected controls, despite overall lower numbers of CD4^+^ T cells in the CSF of PWH (Figure 4D). In contrast, gene expression pathways in CD8^+^ T cells in both the CSF and the blood differed significantly between PWH and uninfected controls, with significant pathways including interferon signaling, Th1 activation, IL-10 signaling, and T cell exhaustion.

We performed an upstream regulator analysis on IPA software to identify the cascade of upstream transcriptional regulators that could explain the observed gene expression changes in our data set, thus further illuminating biological activities that differentiate T cells from PWH and uninfected controls. Upstream regulator analysis revealed that the Th1 cytokines IL-2 and IFN-γ as well as IL-15 were among the most significant upstream regulators that differentiated CSF T cells in PWH from CSF T cells in uninfected individuals.

### PWH on suppressive ART demonstrate persistent perturbations in selected CNS cytokines.

**Figure 5:**
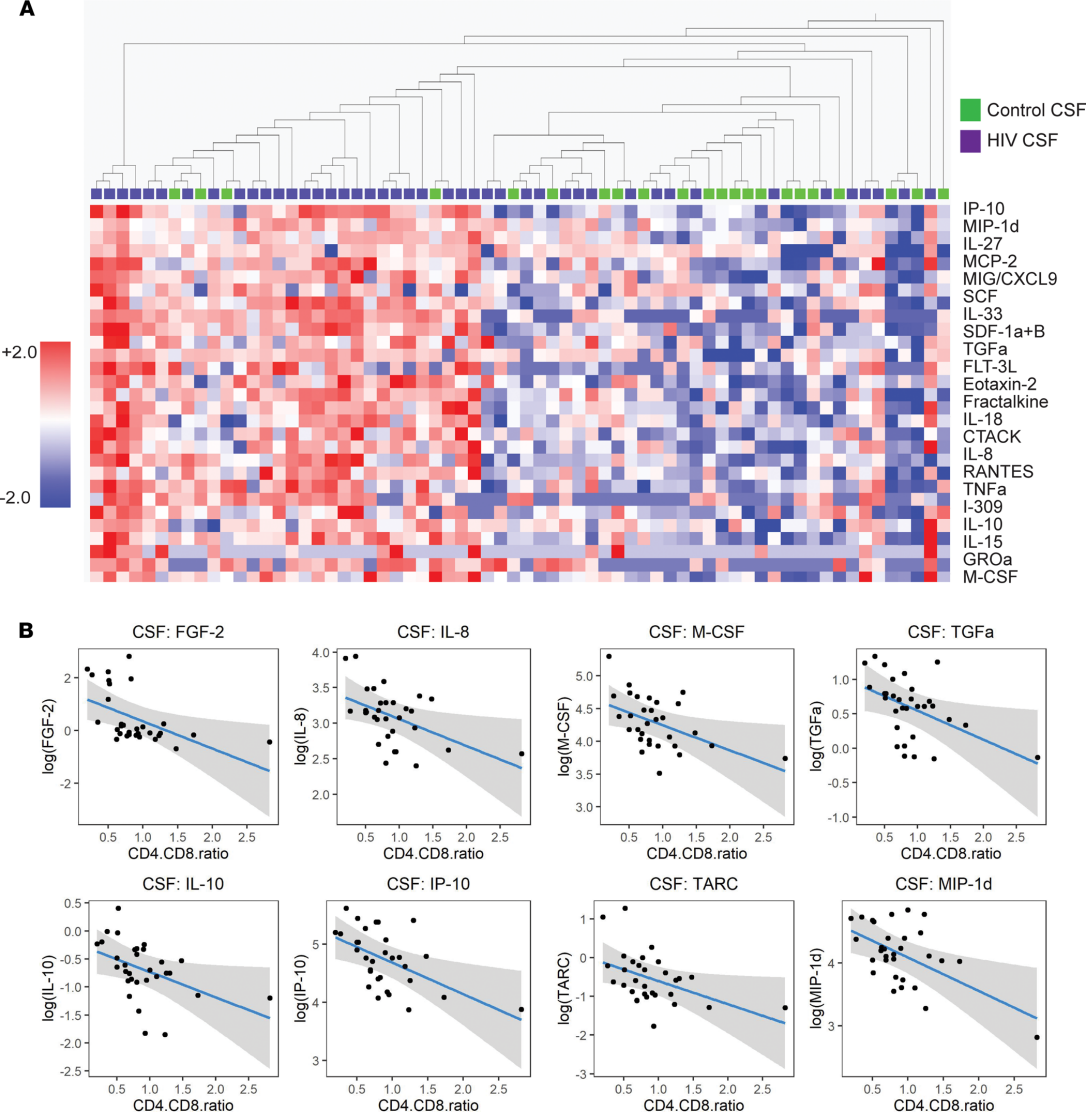
CSF inflammatory cytokines and chemokines are dysregulated in PWH despite ART. (**A**) Heatmap demonstrating cytokine levels in the CSF of PWH (purple) compared with uninfected controls (green). Shown is the list of cytokines for which levels in PWH are significantly different from uninfected controls (*q* < 0.05). Cytokines are ordered by significance (*q* value), and participants are ordered by hierarchical clustering. Adjusted *P* value (*q*) computed by paired *t* tests and FDR controlled using Benjamini, Krieger, and Yekutieli. (**B**) Linear regression analysis demonstrating the effect of the CD4/CD8 T cell ratio on CSF cytokines in PWH after controlling for age, race, sex, and history of substance use disorder. *n* = 44 PWH, *n* = 22 uninfected controls.

Since single-cell analyses revealed immune alterations in the CNS of PWH on suppressive ART compared with uninfected controls, we performed cytokine analysis on the entire cohort, to validate the proteomic effects of the transcriptional changes we observed through scRNA-Seq. We assessed levels of 71 cytokines and chemokines in paired plasma and CSF supernatant from PWH (*n* = 44) and uninfected controls (*n* = 22). An unsupervised heatmap constructed from soluble cytokines and chemokines revealed marked changes in the CSF of PWH compared with uninfected controls (Figure 5A). Overall, compared with controls, PWH demonstrated significantly elevated levels of 21 cytokines and chemokines in the CSF (*P* adjusted < 0.05) (Supplemental Figure 3A), including markers of monocyte chemotaxis and activation (MCP-2/CCL8, SDF-1a/CXCL12, MIP-1d, IP-10/CXCL10, and MIG/CXCL9) as well as TNF-α. In the plasma, only 2 cytokines (MCP-1/CCL2 and MIG/CXCL9) were significantly altered in PWH compared with uninfected controls (Supplemental Figure 3B). Within the HIV cohort, we used a linear regression model to identify CSF cytokines that correlated with the CD4/CD8 ratio (Figure 5B).

We next assessed for cytokine levels that were perturbed in PWH compared with uninfected controls after controlling for age, sex, race, and a history of substance use disorder (Supplemental Tables 2 and 3). We found that HIV infection associated with significant elevations in 15 cytokines in the CSF, including members of the IL-1 proinflammatory cytokine family (IL-1β, IL-33, and IL-18), MIG/CXCL9, MCP-2, and, consistent with prior studies (22, 34–36), IP-10 (*P* < 0.05). Overall, cytokine analysis identified Th1 cell–derived pathways, including the upregulation of IL-1 cytokines, as prominent in the CNS of PWH, and further validated our single-cell transcriptome results, which suggested persistent Th1-mediated activation of CD8^+^ T cells in the CNS of PWH, despite suppressive ART.

## Discussion

Although widespread adoption of ART to suppress viral replication has led to markedly decreased rates of HIV-associated dementia, PWH continue to demonstrate higher-than-expected rates of neurological complications, including mild cognitive impairment, which may be due to both virally mediated and immune-mediated toxicities (37). We utilized recent advancements in single-cell genomics to find evidence for central memory CD4^+^ T cells in the CNS containing HIV transcripts despite apparent control of systemic viral replication. This cell type was previously demonstrated to be preferentially infected by HIV in the blood (38). We did not find evidence for infection of microglia-like cells in the CSF. To our knowledge, this is the first study to detect HIV transcripts in CSF T cells using scRNA-Seq and provides important information on the identity of CNS viral reservoirs in PWH on ART.

We found a higher frequency of HIV-1–producing T cells in the CSF than in the peripheral blood. This is consistent with our prior finding that normalized HIV DNA in CD4^+^ T cells from CSF was higher than in blood in most donors (39). The higher burden of HIV-1 RNA–producing cells in the CSF, compared with blood, may reflect preferential trafficking of infected T cells into the CNS, or local replication of infected cells within the CNS compartment. Further analyses, including TCR repertoire analyses, will be needed to assess whether T cells harboring HIV transcripts in the CSF represent CNS-specific infected clones, or whether they reflect trafficking of infected cells from the periphery into the CNS. Moreover, while the presence of viral transcripts within individual CSF cells suggests an active CNS reservoir, further studies are needed to assess whether these infected cells contain full-length mRNAs and whether they contain RNA elements critical for HIV-1 replication.

We further identified unique host cellular features in CNS immune cells by using single-cell differential abundance analysis and a machine learning tool, STG. We found more abundant Th1 cells and CD8 effector T cells in the CSF of PWH compared with controls and found a corresponding increase in Th1-derived cytokines in the CSF, suggesting that persistent Th1-mediated activation of CD8^+^ T cells in the CNS may contribute to neuropathogenesis during chronic HIV infection. Overall, despite largely normal CSF clinical parameters (i.e., no pleocytosis or elevated CSF protein), multiplex cytokine analysis revealed a markedly abnormal immune milieu in the CNS of PWH, including elevations in the CSF, but not the plasma, TNF-α and IL-12, which have previously been found to be elevated in the CSF under neuroinflammatory conditions (40, 41). IL-12 and the IL-12 family member IL-27 are produced in the CNS by myeloid cells, including microglia, and promote the differentiation of Th1 cells (42–44). Their elevation in the CSF of PWH adds further evidence to the concept of chronic microglial activation in PWH despite suppressive ART. Further studies are needed to understand whether these CSF cytokine elevations are abrogated in PWH who initiate ART during acute infection and who have been postulated to have lower levels of CNS immune activation and neuronal injury than PWH who initiate ART during chronic infection (45–47). Moreover, the cell surface marker CD204, which we identified as a reliable marker for microglia-like cells in the CSF, can aid in future studies of neuroinflammation over the course of HIV infection and its treatment, given the importance of microglia on HIV neuropathogenesis.

When examining the cellular makeup of CSF immune cells in PWH compared with controls, we found that, overall, the cellular composition in the CSF was similar between PWH and uninfected controls, with PWH displaying higher frequencies of CD8^+^ and lower frequencies of CD4^+^ T cells, consistent with prior CSF analyses by flow cytometry (48). However, by using DA-seq, a robust method for detecting cell populations whose frequencies differ between 2 conditions and that may not be detected by standard cluster-based methodologies that are typically used in scRNA-Seq studies (31), we found a subpopulation of Th1 cells, as well as the presence of a population of cytotoxic CD8^+^ T cells, not found in the CSF of uninfected controls. This finding was confirmed by transcriptomic analyses, which identified CD8^+^ T cells in the CSF of PWH as transcriptionally distinct from CD8^+^ T cells in the CSF of uninfected controls, in contrast to CSF CD4^+^ T cells, which were transcriptionally similar in PWH compared to controls. Taken together, these results suggest a model in which Th1-polarized CD4^+^ T cells aid in the activation of CD8^+^ effector T cells in the CNS during chronic HIV infection.

This study has limitations. The presence of gene expression differences does not necessarily indicate a causal relationship with clinical outcomes. Although we assessed neuropsychological performance in PWH and controls, revealing similarities between our 2 groups, our sample size was too small to identify associations between cognitive impairment and immune patterns or detection of HIV transcripts. Further studies are needed to assess for associations between the immunological and viral characteristics we observed and clinical phenotypes. Furthermore, our results should be interpreted with the caveat that CSF biomarkers imply but do not mirror biomarkers within the brain parenchyma, though distinctions between CSF and peripheral blood suggest that the CSF affords a window into a CNS-specific immune milieu in PWH. Overall, these findings demonstrate the power of advanced genomic and bioinformatic approaches to identify rare infected cells in the CNS, as well as CNS-compartmentalized immune perturbations in PWH on ART.

## Methods

### Human research participants.

PWH participating in the study were on stable ART with plasma HIV RNA levels < 20 copies/mL for >1 year. None had active neurological disease or other infection. Controls were recruited from the surrounding community for research sampling and had laboratory confirmation of negative HIV testing via fourth generation ELISA testing as well as clinical screening for any confounding neurological conditions. All participants consented to large-volume lumbar puncture (up to 30 cc CSF removed) and blood draw for research purposes. The age and sex of all human research participants, as well as sample size, are listed in Table 1.

### CSF and blood processing for scRNA-Seq.

Fresh CSF and blood were processed within 1 hour of collection. CSF was centrifuged at 300*g* for 5 minutes at 4°C. Supernatant was removed and cells were resuspended for 10x Genomics processing. PBMCs were isolated via Ficoll gradient with Sepmate tubes and resuspended in PBS-BSA for 10x processing. Approximately 5000 cells per sample (blood or CSF) were processed using the Chromium (10x Genomics) 3*′* single cell expression system v2.0. Samples were sequenced on Illumina HiSeq 4000 or Illumina HiSeq2500 at an average depth of 60,000 reads per cell.

### scRNA-Seq and differential abundance analysis.

Blood and CSF samples from PWH and HIV-uninfected control participants were analyzed using Seurat v3.0 (23, 49) with R version 3.4.2. First, gene-cell expression matrices from Cell Ranger for each sample were filtered based on the following criteria: i) cells with fewer than 500 genes or more than 2000 genes expressed were removed; ii) cells with more than 8% mitochondria transcript content were removed; and iii) cells with more than 1.25% ATP transcript content were removed. Subsequently, samples from only blood, only CSF, only HIV, or only uninfected controls were merged separately. For each merged data set, we applied log transformation after library size normalization, scaling, variable feature selection, and principal component analysis. The top 20 principal components were used for computing t-distributed stochastic neighbor embedding coordinates for visualization, as well as differential abundance analysis to identify most DA cell subpopulations between different states/tissues in the merged data (BLD: HIV vs. control, CSF: HIV vs. uninfected, uninfected: CSF vs. BLD, HIV: CSF vs. BLD) with the tool DA-seq (31). To detect HIV viral transcripts in 10x Genomics scRNA-Seq data from blood and CSF, a single custom reference with human GRCh38 for protein coding genes and HIV viral consensus complete genome sequence HXB2 (GenBank: K03455.1) was used to create a custom reference with cellranger mkref from the 10x Genomics Cell Ranger pipeline.

### Feature selection by STG.

The problem of feature selection is highly related with the task of differential expression analysis (DEA). DEA is typically based on univariate tests and seeks for genes that are typical for certain medical conditions. On the other hand, feature selection methods can identify combinations of genes that allow us to differentiate among the different medical conditions.

Given a set of measurements 
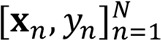
. (pairs of single-cell vectors with labels indicating medical condition), the task of feature selection can be formulated as the following minimization problem:

 (Equation 1)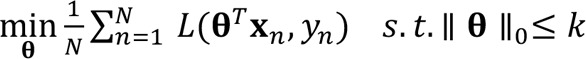,

where *L* is a loss function and θ are the model parameters and *k* is the number of selected features. The *ℓ*_0_ norm does not allow for a solution using continuous optimization schemes. One popular solution is to replace this norm with the *ℓ*_1_ norm, known as the Least Absolute Shrinkage and Selection Operator (50).

In this work, we use STG (51), a recently proposed feature selection method. STG is based on relaxed Bernoulli variables *z_d_*, where ℙ(*z_d_* = 1) = π*_d_*, *d* = 1..., *D,* and *D* is the total number of genes. Then, the parameters of the gates *π_d_* are trained along with the model parameters θ by minimizing over the following loss:

 (Equation 2)
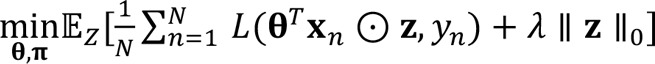


The product ʘ is an element-wise multiplication, and λ is a regularization parameter controlling the number of selected genes. Note that since 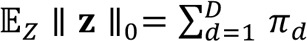, this loss is differentiable and can be optimized using standard optimization schemes, such as gradient descent. To identify genes that are typical to PWH compared with uninfected controls, we optimize over Equation 2 with a binary classification loss defined by


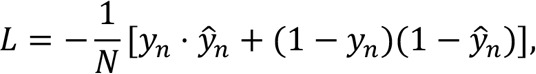
,

where *ŷ_n_* and 1 – *ŷ_n_* represent the predicted probabilities that the *n*th cell belongs to a person with HIV or uninfected patient, respectively. To force that *ŷ_n_* ∈ [0,1], we use a sigmoid activation:


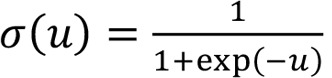


Therefore, the predicted probabilities are defined as 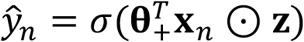.

We first trained the model to identify genes whose variable expression differentiates T cells from a person with HIV versus from an uninfected control. We used a training set of peripheral blood T cells from 4 PWH and 3 uninfected controls and tested the model on the remaining pair of individuals (1 with HIV and 1 uninfected). We repeated this test training in a leave-two-out cross-validation process.

### Gene pathway analysis.

Pathway and upstream regulator analysis was done using IPA (QIAGEN). Significantly differentially expressed genes were mapped to its corresponding gene object in the IPA knowledge base. The overlap between these and relevant pathways or upstream regulators was calculated by the overrepresentation method using the Fisher’s exact test and corrected for the Benjamini-Hochberg FDR (50).

### CITE-seq.

Antibody-oligonucleotide conjugations (“CITE-seq antibodies”) were performed using iEDDA-click chemistry according to a previously described protocol (52). Oligonucleotides (100 nmol scale) were ordered from IDT, amine-modified, reacted with TCO linker (Click Chemistry Tools), desalted using Bio-Rad Micro Bio-Spin P-6 columns, and assessed using a NanoDrop (Thermo Fisher Scientific) and Bioanalyzer (Agilent Technologies). Purified antibodies for immune surface markers for conjugation were obtained from BioLegend, labeled with mTz-PEG4-NHS (Click Chemistry Tools), quenched with glycine, and filtered. The following antibodies were used: Anti-CD16:3G8 (BioLegend, catalog 302001), Anti-CD14:63D3 (BioLegend, catalog 367101), Anti-CD11b:ICRF44 (BioLegend, catalog 301302), Anti-CD1c:L161 (BioLegend, catalog 331501), Anti-CD163:GHI/61 (BioLegend, catalog 333602), and Anti-CD204:7C9C20 (BioLegend, catalog 371902). Labeled antibody and oligonucleotide were conjugated overnight, quenched, and verified using nonreducing SDS polyacrylamide gel. For CITE-seq staining, CSF was centrifuged at 300*g* for 5 minutes at 4°C to form a cell pellet. The cell pellet was resuspended in 100 μL staining buffer (BD Stain Buffer [FBS], catalog 554656) and 10 μL of blocking reagent (Human BD Fc Block, catalog 564220). CITE-seq antibodies were hybridized to cells following the protocol previously described (28), prior to proceeding with 10x Genomics for scRNA-Seq.

### Neuropsychological testing.

Neuropsychological testing was conducted across the following domains (measures): language/premorbid function (WRAT-4 Reading), executive function (Trail making B, Stroop interference, Letter fluency, Categories), speed of information processing (WAIS-III digit symbol, Stroop Color, Trail A), attention/working memory (WAIS-III symbol search, Stroop Word), verbal learning (Hopkins Verbal Learning Test Revised [HVLT-R] learning trials), verbal memory (HVLT-R retention and recognition), fine motor skills (Grooved Pegboard bilateral), and gross motor (timed gait) (53). All measures were normalized according to the participant’s age, while specific tests were normalized by the education level, sex assigned at birth, and race. The 15 individual test *z* scores were computed by subtracting the raw test score from the demographically corrected normative score and then dividing by the normative standard deviation. The resultant *z* scores were evaluated by averaging the scores of the measures within cognitive domains and all the tests into a total *z* score. Positive scores denote better-than-average performance, while and negative scores reflect impaired performance.

### Cytokines.

CSF supernatant and undiluted ethylenediaminetetraacetic acid plasma were frozen after processing cells for scRNA-Seq. Frozen samples were shipped to Eve Technologies on dry ice and were profiled using the 71-Plex Discovery Assay (Human Cytokine Array/Chemokine Array 71-Plex Panel; catalog HD71, Eve Technologies) according to the manufacturer protocol. For the Discovery Assay, a protein standard consisting of purified cytokines at known concentrations was included in each batch run; absolute concentrations were calculated from the standard curve and reported as pg/mL. Fluorescence intensity values were detected for 59 proteins in the CSF and for all 71 proteins in the plasma (mean > 0 pg/mL). Three cytokines (Eotaxin, MCP-3, PDGF-AA) measured in the CSF were noted to have multiple values flagged by Eve Technologies as having “low bead count or no beads”. These 3 cytokines were removed from the data. All values reported as “OOR<”, defined as out of range below the 4 or 5 parameter logistic standard curve, were replaced with “0,” per the manufacturer protocol. Additionally, all instances of “OOR>”, defined as out of range above the 4 or 5 parameter logistic standard curve, were replaced with the highest observed concentration from the standards, per manufacturer protocol.

### FACS.

All CSF and blood samples were processed for FACS within 4 hours of collection. For CSF, the entire cell pellet, obtained from centrifuging 25–30 cc of CSF for 8 minutes at 350*g*, was stained. For PBMCs, approximately 1 million cells were stained per experiment. Antibody cocktails consisted of BV605-conjugated anti-CD4 (OKT4) (BioLegend; catalog 317437), FITC-conjugated anti-CD8 (RPA-T8) (Thermo Fisher Scientific, catalog BDB564526), PE-conjugated anti-CD204 (7c9c20) (BioLegend; catalog 371903), PE-Cy7–conjugated anti-CD20 (2H7) (BioLegend; catalog 302311), APC-conjugated and eFluor780-conjugated anti-CD3 (UCHT1) (Invitrogen, catalog 47-0038-41), and AmCyan-conjugated eFluor506 Live/Dead (eBioscience catalog 65-0866-14). PBMCs were resuspended in Brilliant Stain Buffer (BD, catalog 563794); CSF cells were resuspended in CSF supernatant. Cells were blocked with Human Fc Block (BD, catalog 564220) for 15 minutes at room temperature. Antibody cocktails were added directly to this mixture for 20 minutes at room temperature. Prior to analysis, cells were washed and resuspended in 1× PBS with 0.5% FBS. Single-color compensation tubes (Ultracomp eBeads) or cells were prepared for each of the fluorophores used and acquired at the start of each flow cytometer run. Samples were sorted using a BD FACSAria II flow cytometer. Samples were gated in FlowJo v10.7.2 according to the schema set out in Figure 2. The number of cells falling within each gate was recorded.

### Data and code.

scRNA-Seq data have been deposited at the National Center for Biotechnology Information’s Gene Expression Omnibus and are publicly available at accession number GSE202410.

All original code is publicly available as of the date of publication. STG code is freely available at https://runopti.github.io/stg/ An R package implementation of DA-seq is freely available at GitHub, https://github.com/KlugerLab/DAseq

### Statistics.

Statistical details can also be found in figures, figure legends, and Methods.

Statistical analysis of demographic data was performed using Microsoft Excel and GraphPad Prism v8.0.1. Comparisons between 2 groups were performed using Mann-Whitney test or χ^2^ test (sex, ethnicity, race, alcoholism, and substance abuse). For cytokine data, unadjusted comparisons between 2 groups (PWH and uninfected controls) were performed using multiple unpaired 2-tailed *t* tests. Statistical significance was determined using *q* < 0.05 (*P* value adjusted) with the FDR controlled using the 2-stage linear step-up procedure of Benjamini, Krieger, and Yekutieli. Heatmaps were generated using Qlucore. Linear regression analyses were conducted to assess for the association between cytokine levels and the presence of HIV infection and clinical or demographic factors (age, sex, race, and history of substance use disorder). Within the HIV cohort, we further assessed for the association between cytokine levels and CD4^+^/CD8^+^ T cell ratio.

### Study approval.

Written informed consent was obtained from all participants under approved human research ethics committee protocols from the Yale Institutional Review Board (HIC 1502015318).

## Author contributions

SS and SFF conceived, planned, and carried out the experiments. JZ, OL, and YK developed and performed bioinformatic analyses. MJC, LCN, and GW assisted with single-cell and CITE-seq experiments. DT assisted with flow cytometry experiments. JC, RC, JY, and MC assisted with participant recruitment and biospecimen handling. YI, HW, RGM, AV, and SM contributed to statistical analyses. SS supervised the project. All authors provided critical feedback and helped shape the research, analysis, and manuscript. SFF wrote the manuscript with input from all authors.

## Supplementary Material

Supplemental data

## Figures and Tables

**Table 1:**
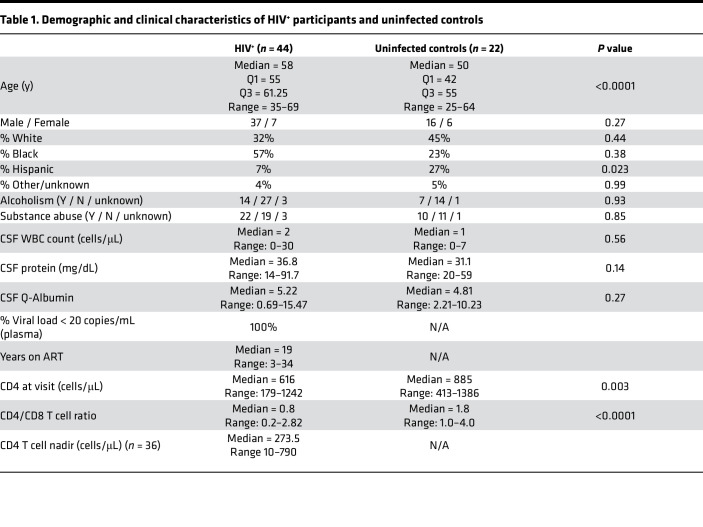
Demographic and clinical characteristics of HIV^+^ participants and uninfected controls

## Version 1 07/08/2022

Electronic publication
