# Supplementary material for: HIV viral transcription and immune perturbations in the CNS of people with HIV despite ART
Source: JCI Insight. 2022 Jul 8;7(13):e160267. doi: 10.1172/jci.insight.160267 (PMC9310520; doi:10.1172/jci.insight.160267)
Supplement: Supplemental data [file jciinsight-7-160267-s100.pdf]

Supplementary Figure 1. Heatmap of the top genes that differentiate clusters shown in Figure 1A.

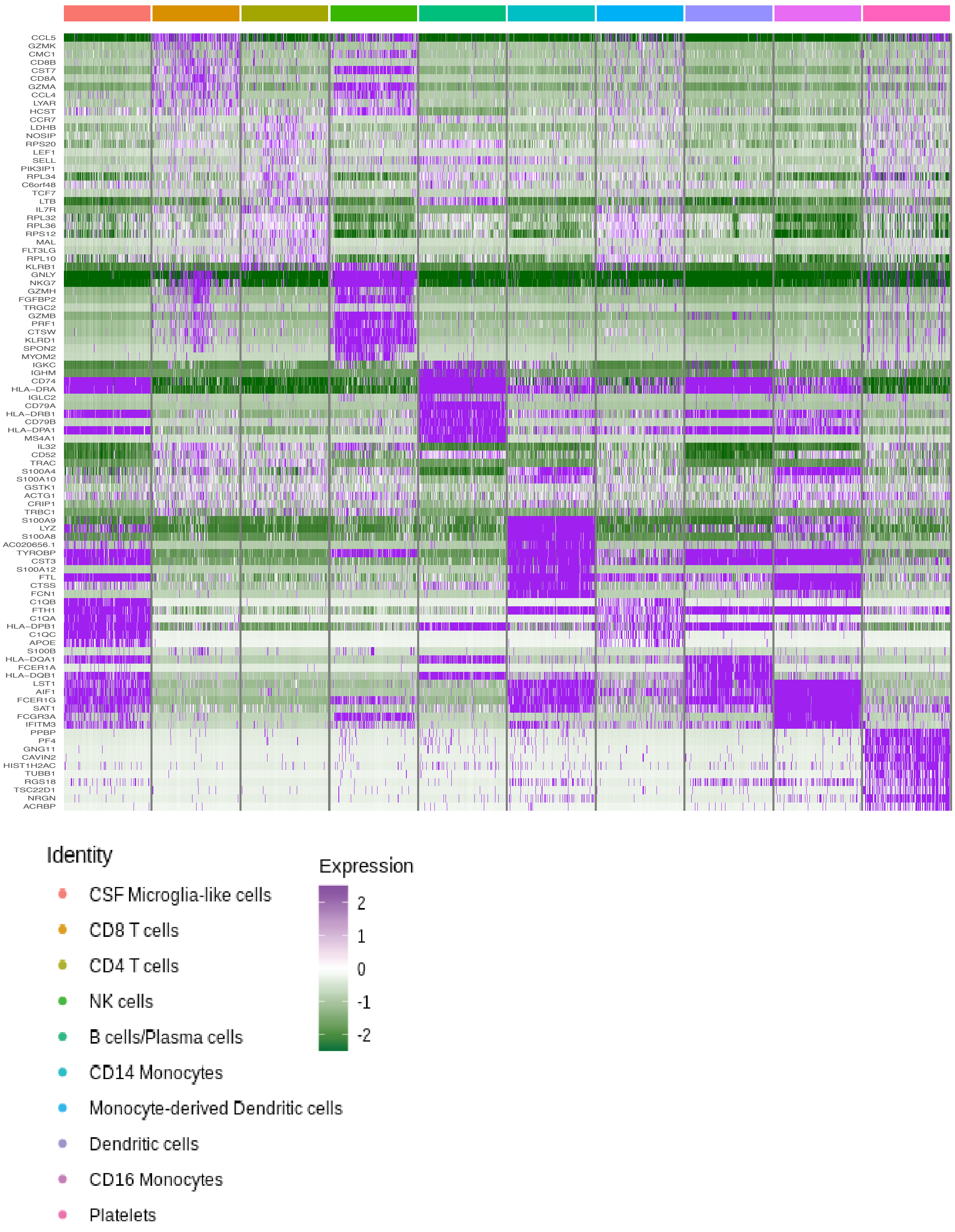

Supplementary Figure 2. Heatmap showing top genes that differentiate the clusters shown in Figure 2A.

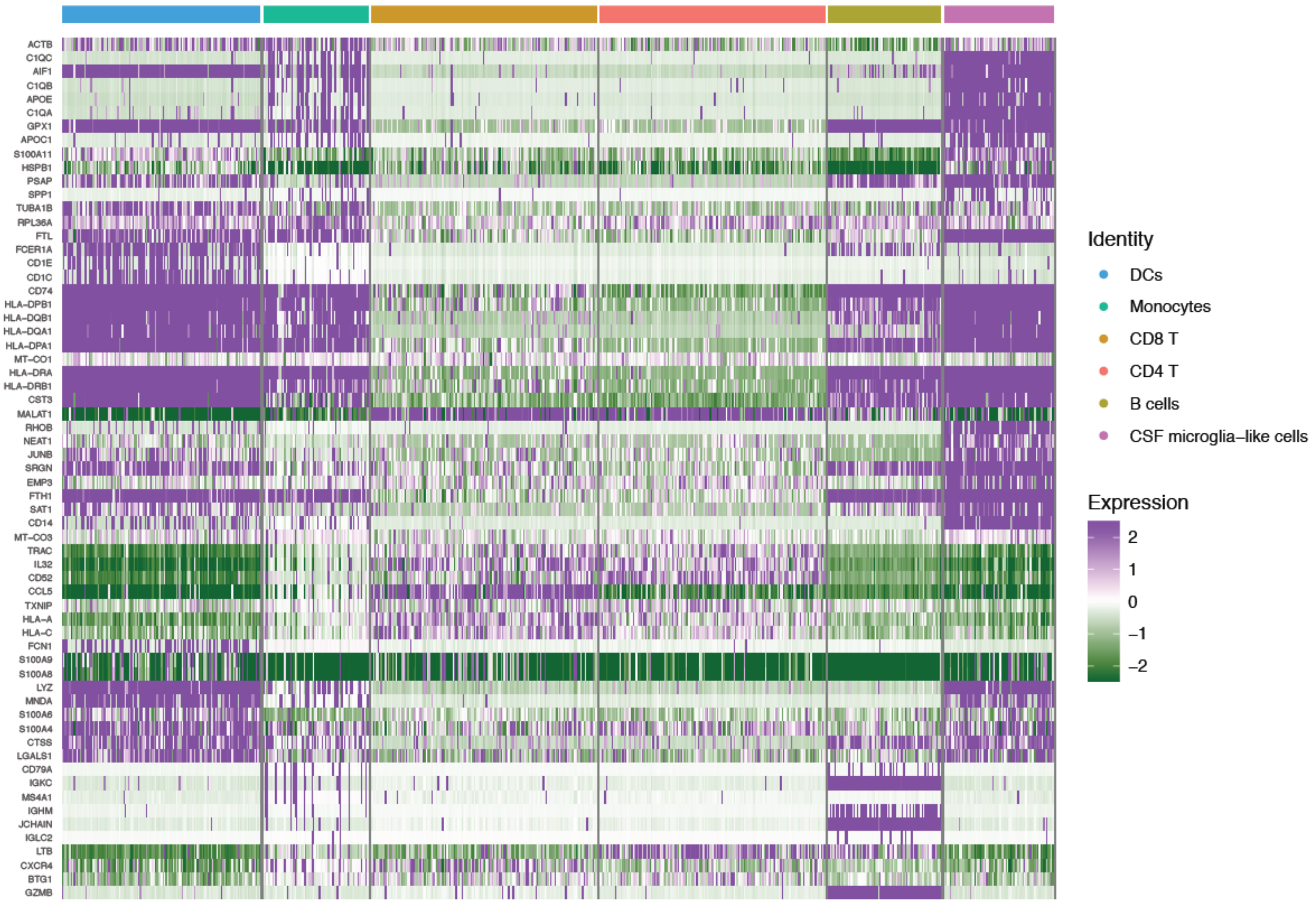

Supplementary Figure 3. Violin plots for individual cytokines elevated in the **CSF (A)** and **Blood (B)** of PLWH (purple) compared to uninfected controls (green) (q<0.05). n= 44 PLWH, n=22 uninfected controls. Adjusted p-value (q) computed by paired t-tests and FDR controlled using Benjamini, Krieger and Yekutieli. Related to Figure 4E.

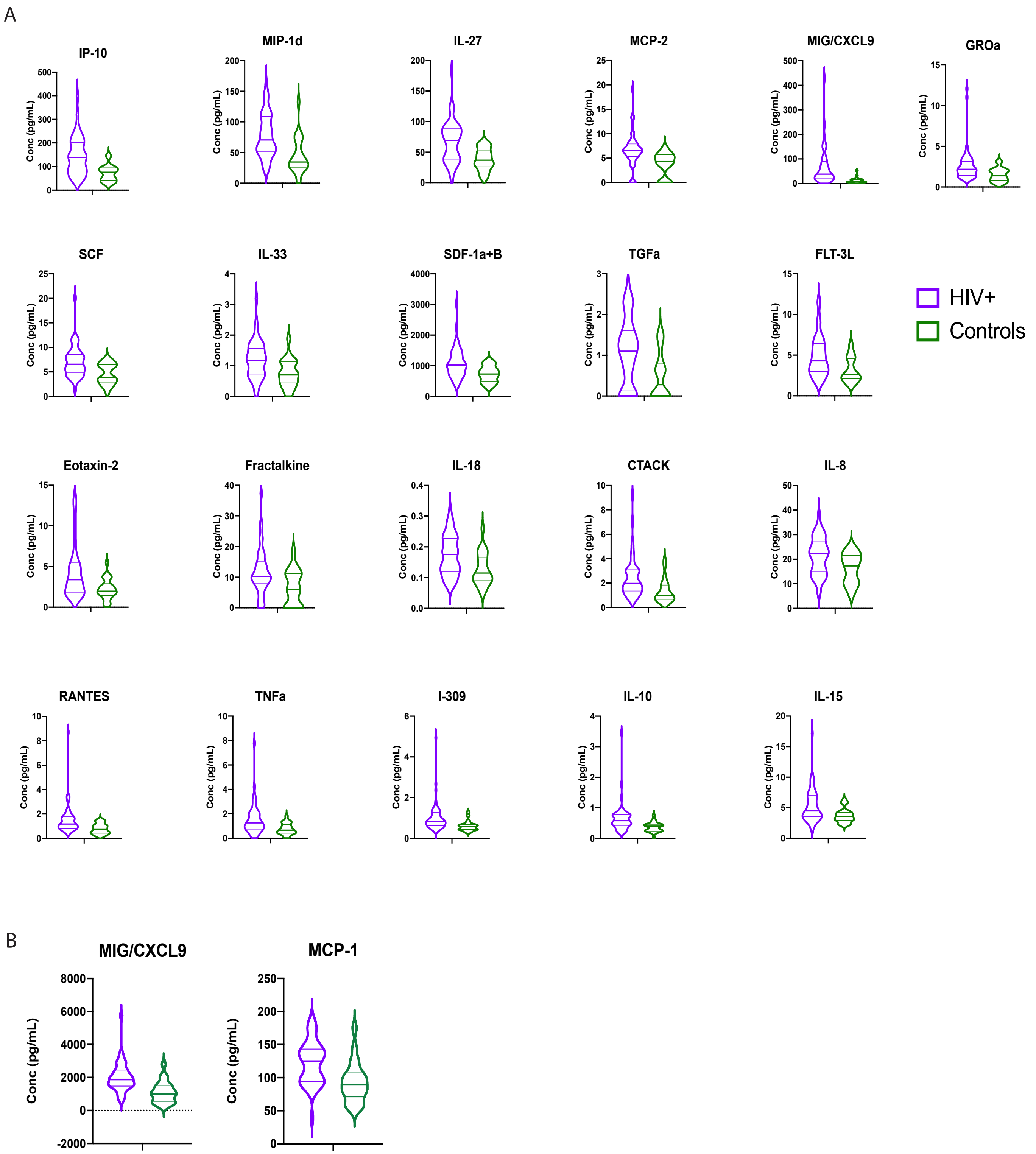

Supplementary Table 1. Characteristics of Research Participants for single cell RNA sequencing (related to figures 1-4)

Subject ID	HIV	Age	Gender	Ethnicity	Race	History of Substance use disorder	History of alcohol use disorder	Plasma HIV Viral Load	CD4 Nadir	CD4 Current	Total Neuropsychiatric Z-score
HIV-37	HIV	36	Male	Non-Hispanic	Black	No	No	<20	78	424	-0.98
HIV-44	HIV	51	Male	Non-Hispanic	White	Yes	No	<20	26	340	N/A
HIV-70	HIV	67	Male	Non-Hispanic	Black	Yes	Yes	Not Detected	Unknown	1242	-1.06
HIV-71	HIV	56	Female	Non-Hispanic	Black	No	No	Not Detected	37	746	-3.53
HIV-72	HIV	57	Male	Non-Hispanic	Black	No	Yes	Not Detected	0	582	-0.62
HIV-73	HIV	60	Female	Non-Hispanic	White	Yes	No	Not Detected	195	596	N/A
CTRL-09	HIV-negative control	54	Male	Hispanic	White	Yes	No			1120	-1.28
CTRL-13	HIV-negative control	55	Male	Non-Hispanic	Black	Yes	No			1052	0.02
CTRL-29	HIV-negative control	59	Male	Non-Hispanic	Black	No	No			833	-0.73
CTRL-31	HIV-negative control	50	Male	Non-Hispanic	Black	Yes	Yes			638	-1.78

Supplementary Table 2. Linear regression analyses demonstrating cytokine levels in the CSF that are significantly affected by HIV status after adjusting for and other factors (age, race, gender, history of substance use disorder). The estimated regression coefficients and their p-values are provided.

Cytokine	Estimate	p-value
GROa	0.03	0.03
IL-18	0.045	0.026
TRAIL	0.341	0.055
IL-10	0.366	0.015
IL-1b	0.368	0.058
IL-33	0.449	0.018
TNFa	0.703	0.009
RANTES	0.769	0.034
IL-12p40	2.122	0.013
MCP-2	3.462	$< 10^{-10}$
MIP-1d	21.674	0.041
IL-27	28.339	0.003
MIG/CXCL9	55.453	0.011
IP-10	66.817	0.001
SDF-1a+B	275.408	0.059

Supplementary Table 3. Linear regression analyses demonstrating cytokine levels in the plasma that are significantly affected by HIV after adjusting for other factors (age, gender, race, history of substance use disorder). The estimated regression coefficients and their p-values are provided.

Cytokine	Estimate	p-value
MIG/CXCL9	1025.055	$< 10^{-10}$
MCP-1	33.485	0.002
IP-10	117.744	0.008
Eotaxin-2	159.017	0.026
BCA-1	18.01	0.038
